# A Rare Case of Infantile Cortical Hyperostosis (ICH) of the Bilateral Tibia or Caffey Disease

**DOI:** 10.7759/cureus.33655

**Published:** 2023-01-11

**Authors:** Hard Tilva, Ankita Kanjiya, Roshan Umate, Mayur B Wanjari, Suhas Tivaskar

**Affiliations:** 1 Department of Obstetrics and Gynaecology, Jawaharlal Nehru Medical College, Datta Meghe Institute of Higher Education & Research, Wardha, IND; 2 Department of Obstetrics and Gynaecology, Sharda Hospital & Research Centre, Surat, IND; 3 Department of Research and Development, Jawaharlal Nehru Medical College, Datta Meghe Institute of Higher Education & Research, Wardha, IND; 4 Department of Radiology, Jawaharlal Nehru Medical College, Datta Meghe Institute of Higher Education & Research, Wardha, IND

**Keywords:** caffey disease, tibia, collagenopathy, anterior cortex, infantile cortical hyperostosis

## Abstract

An inflammatory collagenopathy of infancy characterized by subperiosteal bone hyperplasia is known as infantile cortical hyperostosis (ICH) or Caffey disease. A 10-day male infant presented to the hospital with leg swelling, excessive crying, and irritability since birth. He was born with the swallowed part of his tibia bone. The X-ray suggested hyperostosis of the bilateral tibia bone involving the anterior cortex, which is more prominent on the right side. The infant was clinically monitored and treated and discharged after the swelling was reduced. Again, he was admitted to the hospital at 10 weeks of life, and a similar thickening appeared on his left tibia. He was administered analgesics and non-steroidal anti-inflammatory drugs (NSAIDs) and discharged under a follow-up schedule. The infant was monitored in the pediatric ward for the next seven days. The swelling and pain completely subsided one and a half weeks after hospitalization, and continued follow-up was suggested until the complete correction of the disease on an outpatient basis. This disease must be recognized and understood, and the clinical-radiological correlation is significant.

## Introduction

Infantile cortical hyperostosis (ICH), also called Caffey disease (CD), Caffey-Silverman syndrome, or Smyth syndrome is a rare condition first explicitly characterized by Caffey and Silverman in 1945 [1]; however, there have been earlier reports in the 1930s. The disease typically starts as a severe febrile sickness with painful swellings across one or more bones and all the symptoms of acute inflammation. Up to three out of every 1,000 newborns under six months old are affected by it [1].

It is a genetic condition identified by an episode of enormous, subperiosteal new bone formation that typically affects the mandible, clavicles, and long bones' diaphysis in infancy. Infantile cortical hyperostosis commonly develops in the first few months of life and is an inflammatory illness [2].

## Case presentation

A male newborn infant was admitted on the 10th day of life to the tertiary care hospital under the neonatal care unit with a history of leg swelling, excessive crying, and irritability since birth. As narrated by his mother, despite having no fever, vomiting, or altered eating patterns, she noted that the infant cried when she gently touched the area. He was born with a swallowed part of his right tibial bone under full-term normal vaginal delivery. A blood test was performed, and it showed a typical result. Also, comprehensive blood tests, including C-reactive protein (CRP), were negative. The family history was nil.

On admission, physical examinations showed the baby had an anxious look, and he revealed bilateral tibial bone swellings with tenderness. The baby immediately cried on palpation and handling of the leg, and tachycardia was associated with crying. The underlying skin's color, texture, and temperature remained unchanged. His growth parameters, immunization history, and developmental milestones conducted at the hospital were all within normal ranges.

**Figure 1:**
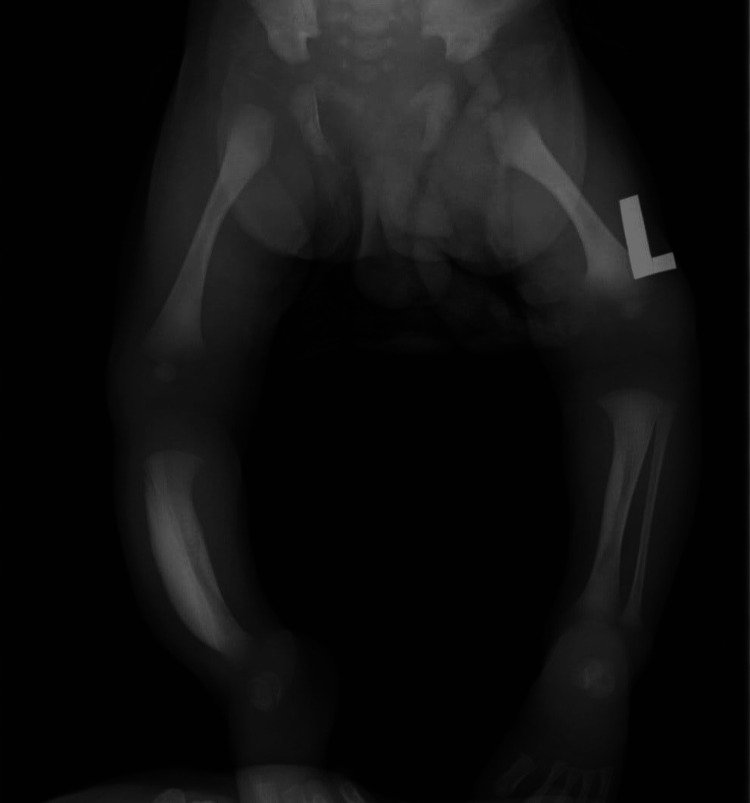
Hyperostosis of the bilateral tibia bone involving the anterior cortex, which is more prominent on the right side

On the third day of life, an X-ray was taken; it was suggestive of hyperostosis of the bilateral tibia bone involving the anterior cortex, which was more prominent on the right side (Figure 1). The infant was clinically monitored for five days. Analgesics and non-steroidal anti-inflammatory drugs (NSAIDs) were administered. He was discharged after the swelling reduced, and regular follow-up visits for the infant were scheduled.

Again, at 10 weeks of life, he was admitted to the hospital, and a similar thickening appeared on his left tibia. It seemed painful, and the newborn was less calm than before. He was started on analgesics (syrup acetaminophen) and anti-inflammatory (ibuprofen) drugs. The infant was monitored in the pediatric ward for the next seven days. After the swelling had reduced, he was discharged and managed on an outpatient basis. The swelling and pain completely subsided one and a half weeks after hospitalization, and continued follow-up was suggested until complete correction of the disease on an outpatient basis.

## Discussion

ICH is usually a self-limited condition of infancy with skeletal abnormalities, soft tissue swelling, fever, irritability, decreased appetite, and reduced movement of the affected bones [3]. Its etiology and pathology are still unknown; a toxic, viral, or bacterial cause doesn't seem likely. Leucocytosis, an elevated erythrocyte sedimentation rate, and high alkaline phosphatase levels are seen in laboratory tests for some patients who are agitated, immobile, and experiencing a temperature increase [4]. Radiography is the most accurate diagnostic procedure in ICH. By using an X-ray, the most usual and diagnostically illustrative findings are obtained. The distinctive feature is the growth of new cortical bone under the areas of swollen soft tissue. No particular laboratory test is available to diagnose ICH [5].

Most cases of Caffey disease are self-limiting, resolve in six to 12 months, and may not require any treatment. However, NSAIDs may be administered in cases of severe symptoms. It has a good effect on the condition. Adverse impact and recurrence are pretty uncommon [6]. It often resolves on its own and exhibits spontaneous inflammatory symptoms. Although their utility is debatable, corticosteroids have been used to manage severe symptoms. There is evidence that non-steroidal anti-inflammatory medicines, usually naproxen, are effective in controlling symptoms [7].

ICH can result in significant bone deformities that may require surgical treatment. Long-term effects could include facial and mandibular asymmetry, occasionally requiring surgical correction [8]. It is crucial to have a high degree of suspicion and be aware of Caffey disease because it is a rare disease that might present with inconsistent clinical symptoms that resemble osteomyelitis. In most cases, a thorough medical history, thorough physical examination, straightforward laboratory tests, and ordinary radiographs are sufficient to make a diagnosis. An MRI can assist in ruling out the most significant differential diagnoses [9]. The prognosis for Caffey disease is often good because it usually resolves by age two. However, the illness might occasionally recur in adolescence or childhood [10].

## Conclusions

ICH, or Caffey disease, affecting newborns and young infants, is a rare and typically self-limiting disorder. Cortical hyperostosis is always seen on radiological tests and may or may not be related to soft tissue edema. This disease must be recognized and understood, and the clinical-radiological correlation is significant. In nations with low resources, like India, avoiding unnecessary studies and treatments depends on people being aware of Caffey's illness, its typical clinical and radiological profile, and its self-limiting nature. Although uncommon and well-understood, the diagnosis of persistent cortical hyperostosis must be invoked in a child who has Caffey syndrome as an infant and exhibits the same clinical signs during adolescence.
